# The regulation of ethylene biosynthesis: a complex multilevel control circuitry

**DOI:** 10.1111/nph.16873

**Published:** 2020-09-12

**Authors:** Jolien Pattyn, John Vaughan‐Hirsch, Bram Van de Poel

**Affiliations:** ^1^ Molecular Plant Hormone Physiology Laboratory Division of Crop Biotechnics Department of Biosystems University of Leuven Willem de Croylaan 42 Leuven 3001 Belgium

**Keywords:** 1‐aminocyclopropane‐1‐carboxylic acid (ACC) metabolism, ethylene biosynthesis, posttranslational regulation, *S*‐adenosyl‐l‐methionine (SAM) metabolism, transcriptional regulation, Yang cycle

## Abstract

The gaseous plant hormone ethylene is produced by a fairly simple two‐step biosynthesis route. Despite this pathway’s simplicity, recent molecular and genetic studies have revealed that the regulation of ethylene biosynthesis is far more complex and occurs at different layers. Ethylene production is intimately linked with the homeostasis of its general precursor *S*‐adenosyl‐l‐methionine (SAM), which experiences transcriptional and posttranslational control of its synthesising enzymes (SAM synthetase), as well as the metabolic flux through the adjacent Yang cycle. Ethylene biosynthesis continues from SAM by two dedicated enzymes: 1‐aminocyclopropane‐1‐carboxylic (ACC) synthase (ACS) and ACC oxidase (ACO). Although the transcriptional dynamics of *ACS* and *ACO* have been well documented, the first transcription factors that control *ACS* and *ACO* expression have only recently been discovered. Both ACS and ACO display a type‐specific posttranslational regulation that controls protein stability and activity. The nonproteinogenic amino acid ACC also shows a tight level of control through conjugation and translocation. Different players in ACC conjugation and transport have been identified over the years, however their molecular regulation and biological significance is unclear, yet relevant, as ACC can also signal independently of ethylene. In this review, we bring together historical reports and the latest findings on the complex regulation of the ethylene biosynthesis pathway in plants.

## Introduction to the ethylene biosynthesis pathway

The volatile hormone ethylene is a major regulator of many developmental and physiological responses in plants (Abeles *et al*., 1992). As a gas, ethylene quickly diffuses from sites of production, where it can be perceived, but is not modified nor metabolised. As such, precise regulation of ethylene biosynthesis is crucial. This is achieved by transcriptional and posttranslational regulation of the ethylene biosynthesis enzymes and the adjacent Yang cycle, and by transport and conjugation of the ethylene precursor 1‐aminocyclopropane‐1‐carboxylic acid (ACC). Although ACC was first identified in plants in the 1950s (Burroughs, 1957; Vahatalo & Virtanen, 1957), only recently new evidence has been gathered which showed that ACC also acts as a signalling molecule in plants, independently of ethylene. ACC has been shown to regulate cell wall metabolism (Xu *et al*., 2008; Tsang *et al*., 2011), guard cell differentiation (Yin *et al*., 2019), vegetative development (Tsuchisaka *et al*., 2009; Vanderstraeten *et al*., 2019) and pollen tube attraction (Mou *et al*., 2020) in Arabidopsis (*Arabidopsis thaliana*). These new insights add weight to the importance of a careful regulation of ACC homeostasis due to its dual role in controlling ethylene biosynthesis and ACC signalling.

**Fig. 1:**
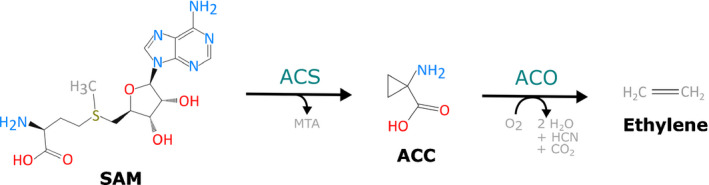
Ethylene biosynthesis progresses from *S*‐adenosyl‐l‐methionine (SAM) via two exclusive enzymatic reactions, catalysed by 1‐aminocyclopropane‐1‐carboxylic acid (ACC) synthase (ACS) and ACC oxidase (ACO).

The ethylene biosynthesis pathway is relatively simple, taking place via only two committed enzymatic reactions (Fig. 1). In the first step, the substrate *S*‐adenosyl‐l‐methionine (SAM) is converted to ACC and 5′‐methylthioadenosine (MTA) by the enzyme ACC synthase (ACS) (Adams & Yang, 1977, 1979; Boller *et al*., 1979). In the second step, ACC is converted to ethylene, CO_2_ and cyanide, by the enzyme ACC oxidase (ACO) (Hamilton *et al*., 1991; Ververidis & John, 1991). The toxicity of the cyanide by‐product is rapidly dealt with by conversion to β‐cyanoalanine (Yip & Yang, 1988) by a group of β‐cyanoalanine synthases (Hatzfeld *et al*., 2000). Because ACS and ACO are the only two enzymes dedicated to ethylene biosynthesis, much of the regulation of overall ethylene production occurs by manipulating transcription, translation and protein stability of these two enzymes. Additionally, metabolic regulation of ethylene production is achieved by ACC homeostasis, which encompasses ACC biosynthesis, transport and conjugation.

## Regulation of the dedicated ethylene biosynthesis enzymes ACS and ACO

ACS and ACO exclusively operate in the ethylene biosynthesis pathway. Both enzymes appear to operate in the cytosol (Boller *et al*., 1979; Reinhardt *et al*., 1994; Chung *et al*., 2002; Hudgins *et al*., 2006), however other studies have suggested that ACO could also be associated with the plasma membrane (Rombaldi *et al*., 1994; Ramassamy *et al*., 1998). ACS belongs to the family of pyridoxal‐5′‐phosphate (PLP) dependent aminotransferases, which require vitamin B6 as a co‐factor for activity. ACO is a member of the 2‐oxoglutarate‐dependent dioxygenase (2OGD) superfamily, which require ferrous iron (Fe^2+^) as the active‐site co‐factor, and 2OG and molecular oxygen as co‐substrates for activity. ACO is unique within this family because it uses ascorbate, not 2OG, as a co‐substrate (Kawai *et al*., 2014). Both ACS and ACO belong to multigene families in most plants (Booker & DeLong, 2015; Houben & Van de Poel, 2019) and their activity can be spatially and temporally separated, allowing precise control of ethylene production. Under normal basal levels of ethylene production, it is generally thought that ACS catalyses the rate limiting step in biosynthesis (Adams & Yang, 1979). However, under certain conditions ACO activity is the rate limiting step (Houben & Van de Poel, 2019). Furthermore, *ACS* and *ACO* show tissue specific expression and localisation patterns (Rodrigues‐Pousada *et al*., 1993; Blume & Grierson, 1997; Wang *et al*., 2005; Datta *et al*., 2015; Park *et al*., 2018), indicating that both ACS and ACO are under tight regulatory control, which takes place at the transcriptional, posttranscriptional and posttranslational level. For more insights on the chemical control of ACS and ACO, we refer to a recent review of Depaepe & Van Der Straeten (2019).

### Transcriptional regulation of ACS

Early work on the discovery of several *ACS* clones in different species (Sato & Theologis, 1989; Nakajima *et al*., 1990; Van Der Straeten *et al*., 1990), revealed that *ACS* is a multigene family which is differentially expressed in plants. For example, differential expression of four tomato (*Solanum lycopersicum*) *ACS* genes was found to be necessary for proper navigation of the transition from autoinhibitory (system I) to autocatalytic (system II) ethylene production during tomato fruit ripening (Barry *et al*., 2000). The first transcription factor identified to regulate expression of *ACS* was the MADS box transcription factor SlRIN, which directly enhances expression of some, but not all, of the tomato *ACS* (Ito *et al*., 2008). Many other transcription factors which regulate expression of *ACS* have been identified in recent years (summarised in Table 1). Both positive and negative regulators of *ACS* expression exist and regulate numerous developmental processes. There is also evidence for direct regulation of *ACS* transcription by ethylene. For example, the tomato ethylene response factor SlERF2/TERF2 was shown to interact with the promoter of *NtACS3* when expressed in tobacco (*Nicotiana tabacum*), inducing its expression (Zhang *et al*., 2009). However, the indirect regulation of ACS by ethylene signalling may be more important. Auxins, cytokinins, brassinosteroids, jasmonates and abscisic acid are all known to regulate ethylene biosynthesis (Yang & Hoffman, 1984; Riov *et al*., 1990; Vogel *et al*., 1998). While hormonal regulation of ethylene biosynthesis has been well documented for decades, the identification of the hormone regulated transcription factors which directly modulate *ACS* expression has been more challenging.

**Table 1:** Transcription factors regulating expression of *ACS*.

Species	Target gene	Transcription factor	Up/downregulated	Biological process	Reference
Arabidopsis	*AtACS2, ACS6*	AtWRKY33	Up	Biotic stress	Li *et al*. (2012)
	*AtACS2*	AtTCP5	Down	Petal development	van Es *et al*. (2018)
	*AtACS7, 9, 11*	AtBES1, AtBZR1	Down	Hormonal crosstalk	Lv *et al*. (2018)
	*AtACS2, 5*	AtERF11	Down	Hormonal crosstalk	T. Li *et al*. (2011)
	*AtACS4, 8*	AtABI4	Down	Hormonal crosstalk	Dong *et al*. (2016)
Tomato	*SlACS2*	SlRIN	Up	Fruit ripening	Ito *et al*. (2008)
Sugarcane	*ScACS2*	ScFBH1‐3	Up	Internode maturation	Alessio *et al*. (2018)
Apple	*MdACS1*	MdMYB10	Up	Fruit ripening	Espley *et al*. (2019)
	*MdACS1, MdACS3a*	MdARF5	Up	Hormonal crosstalk	Yue *et al*. (2020)
	*MdACS1*	MdMYC2	Up	Hormonal crosstalk	T. Li *et al*. (2017)
Kiwi	*AdACS1*	AdNAC2, 3	Up	Hormonal crosstalk/fruit ripening	Wu *et al*. (2020)
Japenese plum	*PsACS1*	PsABI5	Down	Hormonal crosstalk/fruit ripening	Sadka *et al*. (2019)

### Posttranslational regulation of ACS

**Fig. 2:**
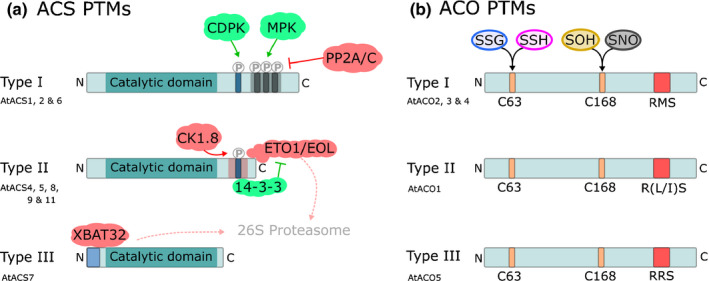
Posttranslational modifications (PTMs) of ACS and ACO. (a) ACS can be divided into three phylogenetically related groups based on the presence of phosphorylation sites for CDPK and MPK (type I), CDPK only (type II) or no C‐terminal regulatory sites (type III). Phosphorylation of type I ACS (CDPK and MPK) positively effects protein stability, while dephosphorylation (PP2A/C) negatively effects protein stability. Phosphorylation of type II ACS by CK1.8 enhances binding of ETO1/EOL, leading to protein degradation. This interaction is repressed by 14‐3‐3 proteins, which bind ACS and destabilises ETO1/EOL. XBAT32 directly binds type III ACS leading to protein degradation. Interactions depicted in green and red are positive and negative regulators of ACS activity, respectively. (b) ACO forms three phylogenetically related groups that can be separated based on the amino acids present in the RXS motif (red box). ACOs share two conserved cysteine residues which have been associated with posttranslational modifications (C63 and C168). Modifications at C63 include *S*‐glutathionylation (SSG) and *S*‐sulfhydration (SSH), whereas modifications at C168 include *S*‐sulfenylation (SOH) and *S*‐nitrosylation (SNO). So far, these cysteine modifications have only been described in type I ACO.

As well as the transcriptional, the posttranslational regulation of ACS is crucial for controlling ethylene production (Booker & DeLong, 2015). The regulation via posttranslational modifications depends on particular residues within the ACS proteins (Fig. 2). While the N terminus and catalytic core of different ACS isoforms are well conserved, there is more variability in the C terminus. Based on the presence of particular sequences at the C terminus, the ACS family can be divided into three major groups: type 1 has target sites for mitogen‐activated and calcium‐dependent protein kinases (MAPK and CDPK respectively), type 2 has target sites for CDPK and E3 ligases, and type 3 has no target sites (for review see Yoon, 2015). Studies from various species have identified kinases that stabilise type 1 ACS by phosphorylation, thereby promoting ethylene production (Tatsuki & Mori, 2001; Joo *et al*., 2008; Li *et al*., 2012; Meng *et al*., 2014). By contrast, dephosphorylation has been shown to promote degradation of ACS, although this is dependent on the ACS type (Skottke *et al*., 2011; Ludwików *et al*., 2014). In some cases, phosphorylation can also destabilise ACS. This was demonstrated by the phosphorylation of Arabidopsis type 2 ACS5 by Casein Kinase 1.8 (CK1.8), which promotes interaction with the E3 ubiquitin ligase ETO1 (Ethylene Overproducer1) and EOL (ETO1‐Like), leading to ubiquitination and consequent degradation of ACS5 via the 26S proteasome (Chae *et al*., 2003; Wang *et al*., 2004; Tan & Xue, 2014). While the C‐terminal domain of type 1 and type 2 ACS is certainly important for regulation of protein stability, a short region of the N‐terminal domain of the Arabidopsis type 3 ACS7 fulfils this role, as it can be marked for degradation via ubiquitination by XB3 orthologue 2 in Arabidopsis (XBAT32; Lyzenga *et al*., 2012; Xiong *et al*., 2014). ACS7 can also be stabilised via the interaction with several members of the Protein Phosphatase 2C family (PP2C’s; Marczak *et al*., 2020). Another mechanism of ACS stabilisation occurs via the interaction with 14‐3‐3 proteins that protects them from degradation (Yoon & Kieber, 2013). In general, ACS protein stability is controlled by developmental and hormonal regulators, including cytokinins, brassinosteroids, auxins, jasmonic acid, abscisic acid, salicylic acid and gibberellic acid (Lee *et al*., 2017).

In addition to posttranslational modifications of ACS, hetero‐ or homo‐dimerisation of different ACS isoforms has been shown to influence enzyme activity. While ACS homodimers show enzymatic activity (Tarun & Theologis, 1998), only heterodimers composed of members from the same phylogenetic branch are active (Tsuchisaka & Theologis, 2004). Hetero‐dimerisation also prolongs the half‐life of some short‐lived ACS isoforms (Lee *et al*., 2017). This results in a great diversity of ACS configurations which is dependent on the exact ratio of particular ACS isoforms in each cell, further allowing fine‐tuning of ACC biosynthesis (Tsuchisaka *et al*., 2009).

### Transcriptional regulation of ACO

Several transcriptional regulators of ACO have been identified from various species. The tomato HD‐ZIP transcription factor SlHB‐1 was the first transcription factor identified to directly regulate expression of *SlACO1* (Lin *et al*., 2008). It was later reported that the ripening regulator RIN can bind promoters and upregulate the expression of both *HB‐1* and *SlACO4* (Martel *et al*., 2011; L. Li *et al*., 2017). Various other classes of transcription factor have been implicated in the direct regulation of *ACO* expression in many species, as covered in a recent review by Houben & Van de Poel (2019). Recently, new regulators of *ACO* expression have been identified and are listed in Table 2. Ethylene has also been shown to directly regulate its own biosynthesis by controlling *ACO* expression, both by positive and negative ERF‐mediated feedback mechanisms (see Table 2). Similar to transcriptional regulation of ACS, hormonal crosstalk by auxins, brassinosteroids, jasmonic acid and abscisic acid is involved in the regulation of *ACO* transcription. For transcriptional regulation, there is some evidence for posttranscriptional regulation of ACO, at the level of mRNA transcript stability. In trifoliate orange, miRNA396b is able to cleave Ptr*ACO* transcripts (Zhang *et al*., 2016), while in tomato several noncoding RNAs and miRNAs were found to target different *ACO* transcripts during ripening (Zuo *et al*., 2020).

**Table 2:** Transcription factors regulating expression of *ACO*.

Species	Target gene	Transcription factor	Up/downregulated	Biological process	Reference
Arabidopsis	*AtACO5*	AtSHYG	Up	Abiotic stress	Rauf *et al*. (2013)
	*AtACO4*	AtBZR1	Down	Hormonal crosstalk	Moon *et al*. (2020)
	*AtACO1*	AtBES1	Up	Hormonal crosstalk	Park *et al*. (2020)
	*AtACO2*	AtABI4	Down	Hormonal crosstalk	Dong *et al*. (2016)
Tomato	*SlACO1*	SlHB‐1	Up	Fruit ripening	Lin *et al*. (2008)
	*SlACO4*	SlRIN	Up	Fruit ripening	L. Li *et al*. (2017)
	*SlACO1*	SlNAC4, 9	Up	Fruit ripening	Kou *et al*. (2016)
	*SlACO3*	SlERF2, TERF2	Up	Seedling development	Zhang *et al*. (2009)
Banana	*MaACO1*	MaERF11	Down	Fruit ripening	Han *et al*. (2016)
	*MaACO1*	MaMADS7	Up	Fruit ripening	Liu *et al*. (2015)
Melon	*CmACO1*	CmEIL1, 2	Up	Fruit ripening	Huang *et al*. (2010)
	*CmACO3*	CmWIP1	Down	Floral development	Chen *et al*. (2016)
Cucumber	*CsACO2*	CsWIP1	Down	Floral development	Chen *et al*. (2016)
Wheat	*TuACO3*	TuMYB46L	Down	Biotic stress	Zheng *et al*. (2020)
Kiwifruit	*AdACO1*	AdNAC6, 7	Up	Fruit ripening	Wang *et al*. (2020)
Apple	*MdACO1*	MdMYC2	Up	Hormonal crosstalk	T. Li *et al*. (2017)
	*MdACS1*	MdMYB10	Up	Fruit ripening	Espley *et al*. (2019)
	*MdACO1*	MdAFR5	Up	Hormonal crosstalk	Yue *et al*. (2020)

### Posttranslational regulation of ACO

The ACO protein family can be divided in three groups (Types 1–3; Fig. 2) based on amino acid sequence similarity of the dioxygenase‐specific conserved RXS motif, important for catalytic activity (Houben & Van de Poel, 2019). While posttranslational regulation of ACS has been well described, information on the posttranslational regulation of ACO has been largely lacking. Dilley *et al*. (2013) identified putative sites for phosphorylation and glycosylation within the ACO protein sequences, although this was not confirmed experimentally. There is more evidence for redox‐specific posttranslational modifications of dedicated ACO cysteine residues (Fig. 2). For example, Arabidopsis ACO1 was documented to be *S*‐glutathionylated, although how this modification affects ACO1 activity remains unknown (Dixon *et al*., 2005; Datta *et al*., 2015). *S*‐sulfhydration of ACO4 was also observed in Arabidopsis (Aroca *et al*., 2017), while in tomato, *S*‐sulfhydration of SlACO1 and SlACO2, was shown to inhibit ACO enzyme activity (Jia *et al*., 2018). Another cysteine modification of ACO, namely *S*‐nitrosylation, was observed in both Arabidopsis and tomato (Hu *et al*., 2015; Gong & Shi, 2019). Recently, two studies have confirmed the importance of redox‐specific cysteine modifications to control ACO activity and structural stability, by performing site‐directed mutagenesis assays (Fournier *et al*., 2019; Tachon *et al*., 2019). Altogether, there is now a growing body of evidence that ACO is redox‐controlled in plants. The exact biological function of these cysteine modifications with respect to ACO activity or stability remain elusive. Another posttranslational modification of ACO is achieved through protein‐protein interactions. A single study in petunia reported that the protein PhGRL2 negatively regulates PhACO1 activity by direct binding with PhACO1 (Tan *et al*., 2014).

## SAM homeostasis influences ethylene production

**Fig. 3:**
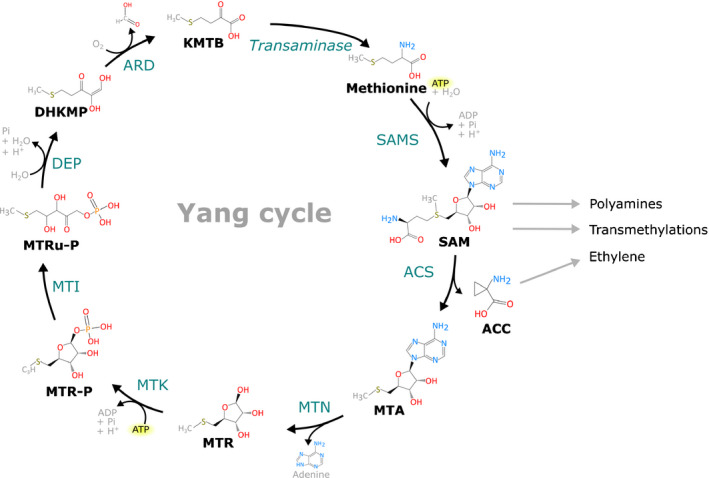
Structural representation of the Yang Cycle, which functions to recycle 5′‐methylthioadenosine (MTA), the by‐product of ACC biosynthesis, back to methionine. The general precursor *S*‐adenosyl‐l‐methionine (SAM) is shared by ethylene biosynthesis and several other pathways. Briefly, MTA is first converted to 5′‐methylthioribose (MTR) by MTA nucleosidase (MTN), releasing adenine (Adams & Yang, 1977). MTR is then phosphorylated by MTR kinase (MTK) into MTR‐phosphate (MTR‐P) and subsequently isomerised by MTR‐P isomerase (MTI) to produce 5′‐methylthioribulose‐1‐phosphate (MTRu‐P) (Kushad *et al*., 1982; Sauter *et al*., 2004; Bürstenbinder *et al*., 2007; Pommerrenig *et al*., 2011). MTRu‐P is then converted to 1,2‐dihidroxy‐3‐keto‐5′‐methylthiopentene (DHKMP) by the enzyme dehydratase‐enolase‐phosphatase (DEP) in a single step, unlike the corresponding reactions in bacteria, fungi and animals which require multiple enzymes (Pommerrenig *et al*., 2011; Sekowska *et al*., 2019). DHKMP is subsequently converted to 2‐keto‐4‐methylthiobutyrate (KMTB) by acireductone dioxygenase (ARD) (Sauter *et al*., 2005). Finally, methionine is formed by the transamination of KMTB via the action of an unknown transaminase (Pommerrenig *et al*., 2011).

Both ACS and ACO rely on the steady supply of SAM as a general precursor, which is derived from methionine via the action of SAM synthetase (SAMS), also known as methionine adenosyltransferase (MAT). Other metabolic pathways also influence the SAM pool (Fig. 3), which pinpoints to a more complex regulation of SAM homeostasis. Aside from its role in ethylene biosynthesis, decarboxylated SAM is involved in the biosynthesis of higher polyamines (spermidine and spermine), molecules that are involved in many aspects of plant growth, development and stress responses (reviewed by Chen *et al*., 2019). Additionally, SAM serves as a universal methyl donor for the largest class of methyltransferases, enzymes which catalyse methylation of a wide range of substrates, such as histones, DNA and RNA to modify transcription and translation (reviewed by Lindermayr *et al*., 2020). Other SAM‐dependent methyltransferases are involved in the metabolism of important specialised plant molecules, such as nicotinamides (Rahikainen *et al*., 2018). New insights also placed SAM homeostasis and transmethylation in relation to virus infections, during which ethylene is also important (Mäkinen & De, 2019). It has been suggested that these different pathways may compete for SAM (Moffatt & Weretilnyk, 2001). However, analysis of ethylene, polyamines and transmethylation levels during ripening of tomato fruit revealed that these pathways are able to function simultaneously, due to an augmentation of the SAM pool during ripening (Van de Poel *et al*., 2013).

*SAMS* genes were shown to be regulated at the transcriptional level by developmental cues and hormonal signalling (Peleman *et al*., 1989; Gómez‐Gómez & Carrasco, 1998). Surprisingly, the effect of ethylene seems to be less important, because *SAMS* expression levels remained unaltered or dropped during developmental stages characterised by high rates of ethylene production such as, pollination of Australian tobacco (*Nicotiana suaveolens*), senescence of carnation (*Dianthus caryophyllus*) petals and climacteric fruit ripening of tomato and peach (*Prunus persica*) (Woodson *et al*., 1992; Roeder *et al*., 2009; Van de Poel *et al*., 2013; Zeng *et al*., 2020). Alternatively, other studies have described the differential regulation of *SAMS* expression in response to stress, in various species, which is often linked with an increase in ethylene production (Kim *et al*., 2015; He *et al*., 2019). Few transcription factors involved in regulating *SAMS* expression have been identified. In tomato, the Auxin Response Factor 6a (SlARF6a) directly inhibits expression of *SlSAMS1*, providing evidence of a direct link between auxin signalling and SAM biosynthesis (Yuan *et al*., 2019).

While mechanistic insights into the transcriptional regulation of *SAMS* is limited, more progress has been made at the level of posttranslational regulation. In Arabidopsis, the gaseous signalling molecule nitric oxide (NO) was shown to inactivate SAMS1 by *S*‐nitrosylation (Lindermayr *et al*., 2006). Interestingly SAMS2 and SAMS3 were not *S*‐nitrosylated, suggesting only SAMS1 is responsible for NO mediated regulation of SAM biosynthesis. It is possible that *S*‐nitrosylation affects ethylene biosynthesis in plants by both targeting SAMS1 and ACOs. In Arabidopsis, SAMS can also be phosphorylated by CPK28, which targets SAMS1‐3 for degradation after phosphorylation (Jin *et al*., 2017). The *cpk28* mutant displays less SAMS degradation leading to higher SAM levels and a higher ethylene production (Jin *et al*., 2017). Another kinase, the receptor‐like kinase FERRONIA, was found to interact with SAMS1 and SAMS2 in Arabidopsis, potentially inhibiting its activity by phosphorylation, leading to a suppression of ethylene biosynthesis (Mao *et al*., 2015). Another protein that directly interacts with OsSAMS1 is Psn11, a protein encoded by the rice dwarf virus (Zhao *et al*., 2017). Psn11 binds and enhances activity of OsSAMS1, increasing ethylene biosynthesis, which aids viral infection (Mäkinen & De, 2019). These studies clearly show that posttranslational modifications of SAMS led to altered ethylene levels, revealing the intimate relationship between SAM homeostasis and ethylene biosynthesis.

## The Yang cycle sustains ethylene biosynthesis

The Yang cycle mainly serves to recycle the precious sulfur moiety from MTA, a side‐product from ACS activity, back to methionine (Fig. 3) (Murr & Yang, 1975; Adams & Yang, 1977; Pommerrenig *et al*., 2011; Sauter *et al*., 2013). The plant Yang cycle shows many similarities, but also some discrepancies, to the methionine salvage cycles found in bacteria, fungi and animals (Murr & Yang, 1975; Adams & Yang, 1977; Sekowska *et al*., 2019).

The activity of the Yang cycle has been linked with the regulation of ethylene production. Early biochemical evidence hinted that the Yang cycle was ample to sustain high rates of ethylene production in ripening apple (*Malus domestica*) discs (Miyazaki & Yang, 1987). It has been shown that several Yang cycle genes are upregulated in plant organs that experience high rates of ethylene production, such as *SlMTN*, *SlMTK* and *SlARD1‐2* in tomato during climacteric fruit ripening (Van de Poel *et al*., 2012) and *OsMTN* and *OsARD1* in rice (*Oryza sativa*) during submergence (Sauter *et al*., 2005; Rzewuski *et al*., 2007). Furthermore, an overexpression line of *OsARD* in rice resulted in increased ethylene production, leading to better submergence tolerance (Liang *et al*., 2019). However, in Arabidopsis, Yang cycle genes (*MTN*, *MTK* and *ARD1,2,4*) are not ethylene regulated, and a *mtk* mutant does not result in lower ethylene levels in dark grown seedlings (Bürstenbinder *et al*., 2007). These observations suggest that the Yang cycle is only important for those plants that naturally produce high levels of ethylene such as ripening tomato fruit or flooded rice plants (Bürstenbinder *et al*., 2007). In Arabidopsis, high levels of ethylene production become relevant during certain pathogen infections. The *Pseudomonas syringae* effector HopAF1 leads to dampened ethylene production, by hijacking MTN activity, resulting in enhanced disease susceptibility (Washington *et al*., 2016). One can speculate that recycling of MTA by the Yang cycle is essential for high rates of ethylene production, or conversely, that high rates of ethylene production stimulate MTA recycling via Yang cycle enzymes. Possibly, the latter is true, as MTA was found to be a weak inhibitor of ACS activity (Miyazaki & Shang, 1987), so removal of MTA via the Yang cycle could prevent inhibition of ACS and sustain ethylene production. Further studies confirmed that MTA is the main trigger for activating the methionine salvage pathway in plants (Sauter *et al*., 2013).

## The regulation of ACC homeostasis through conjugation

**Fig. 4:**
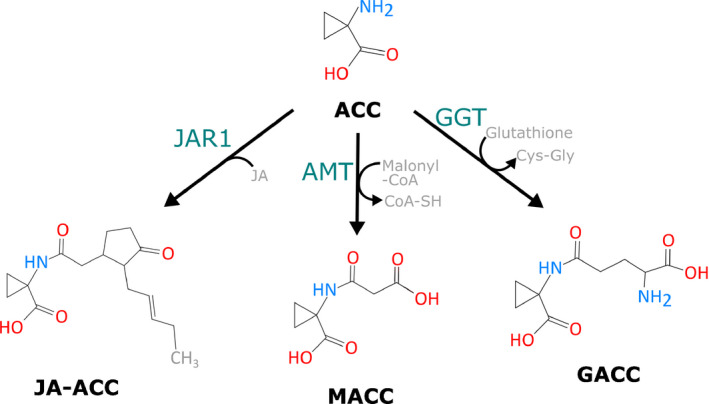
Structural representation of 1‐aminocyclopropane‐1‐carboxylic acid (ACC) conjugates. Malonyl‐1‐aminocyclopropane‐1‐carboxylic acid (MACC), the most abundant ACC conjugate, is formed by ACC‐N‐malonyl transferase (AMT). Jasmonyl‐1‐aminocyclopropane‐1‐carboxylic acid (JA‐ACC) is formed by Jasmonic Acid Resistance 1 (JAR1). γ‐glutamyl‐1‐aminocyclopropane‐1‐carboxylic acid (GACC) is formed by γ‐glutamyl transpeptidase (GGT).

ACC serves as the unique precursor of ethylene biosynthesis and recent insights pinpoint this small cyclopropane to be a signalling molecule independent of ethylene (reviewed by Van de Poel & Van Der Straeten, 2014; Polko & Kieber, 2019). This implies that the ACC pool is strictly regulated to serve both ACC signalling and ethylene biosynthesis. Plants have evolved an elegant mechanism to control the pool of active signalling molecules by derivatisation and catabolism. While other hormonal pathways are known to have several conjugates of their active compounds, ethylene has none, as gaseous modifications are unlikely to take place. Conjugation predominately occurs at the level of ACC and, so far, three derivatives have been identified: malonyl‐ACC (MACC), glutamyl‐ACC (GACC) and jasmonyl‐ACC (JA‐ACC) (Fig. 4). Compared with other hormonal pathways this is a rather low number of derivatives, so perhaps other ACC conjugates await to be discovered.

### MACC: the most abundant ACC derivative

MACC was identified as a first ACC derivative in buckwheat (*Fagopyrum esculentum*) seedlings (Amrhein *et al*., 1981) and wheat (*Triticum aestivum*) leaves (Hoffman *et al*., 1982). MACC is formed by ACC‐N‐malonyl transferase (AMT), which transfers a malonyl group from malonyl‐Co‐A onto ACC releasing Co‐A (Fig. 4). Besides malonyl‐Co‐A, a second acyl donor, namely succinyl‐Co‐A, is able to *in vitro* conjugate ACC, albeit less efficiently compared with malonyl‐Co‐A (Benichou *et al*., 1995; Martin & Saftner, 1995). MACC is typically present in a five‐ to 10‐fold higher concentration compared with ACC (Liu *et al*., 1985a; Philosoph‐Hadas *et al*., 1985; Sarquis *et al*., 1992; Van de Poel *et al*., 2012), making it the major conjugate of ACC (Liu *et al*., 1983; Hoffman *et al*., 1983a; Peiser & Yang, 1998). There is also evidence that plants can convert MACC back into ethylene (Yang *et al*., 1982; Hoffman *et al*., 1983a; Matern *et al*., 1984; Jiao *et al*., 1986; Hanley *et al*., 1989), which seems to be tissue specific and age dependent (Matern *et al*., 1984; Yin *et al*., 2019). The enzymatic nature of MACC catabolism and whether or not it operates via the reconversion towards ACC, remain to be uncovered.

Shortly after the discovery of MACC, multiple research groups partially purified AMT from mung bean (*Vigna radiata*) hypocotyls (Kionka & Amrhein, 1984; Su *et al*., 1985; Guo *et al*., 1992; Benichou *et al*., 1995; Chick & Leung, 1997), tomato (Martin & Saftner, 1995) and chick‐pea (*Cicer arietinum*) seeds (Martínez‐Reina *et al*., 1996). Initially it was assumed that AMT was a single monomeric enzyme, however Table 3 shows that different studies reported different molecular masses and enzyme characteristics for AMT. This observation suggests that perhaps multiple isoforms of AMT exist. Despite all these efforts, the actual AMT genetic sequence remains unidentified and certainly blurs our insight into understanding the regulation of ethylene biosynthesis through ACC sequestering into MACC.

**Table 3:** Enzyme characteristics of partially purified AMT enzymes from different plant species.

Species	Molecular mass (kDa)	*K*_m_ for ACC (µM)	*K*_m_ for malonyl‐CoA (µM)	Optimal *T* (°C)	Optimal pH	Reference
Mung bean	‐	170	250	40	8	Kionka & Amrhein (1984)
Mung bean	‐	150	500	35	8	Su *et al*. (1985)
Mung bean	50–55	500	200	50	8	Guo *et al*. (1992)
Mung bean	36	68	74	40	8.5	Benichou *et al*. (1995)
Tomato	38	500	100	‐	8–8.5	Martin & Saftner (1995)
Chick‐pea	54	400	90	40	7.5–8	Martínez‐Reina *et al*. (1996)
Mung bean	40	66.7	40	45	‐	Chick & Leung (1997)

AMT activity seems to be controlled in part by ethylene itself. It was shown that ethylene stimulates MACC formation in nonsenescing tobacco leaf discs (Philosoph‐Hadas *et al*., 1985) and preclimacteric tomato and grapefruit (Liu *et al*., 1985a; Martin & Saftner, 1995). We hypothesise that MACC formation is enhanced in certain tissues where high rates of ethylene production are not yet desirable by scavenging free ACC to prevent ethylene synthesis. Conversely, certain tissues that produce high amounts of ethylene also show high MACC levels. For example, MACC levels were found to increase during ripening of tomato and apple fruit (Peiser & Yang, 1998; Van de Poel *et al*., 2012), senescing of carnation flowers (Hanley *et al*., 1989), cocklebur (*Xanthium strumarium*) germination (Satoh & Esashi, 1984) and mechanical pressure (Sarquis *et al*., 1992). These observations allowed us to conclude that ethylene triggers MACC formation in conditions in which ethylene productions needs to be repressed or activated.

MACC is likely to be synthesised in the cytosol, and can accumulate in the vacuole (Bouzayen *et al*., 1988). These observations suggest that MACC is transported across the tonoplast, presumably requiring specific active (ATP‐consuming) transporters (Bouzayen *et al*., 1988). Once MACC has reached the vacuole, it probably remains there (Bouzayen *et al*., 1988), leading to the idea that MACC is an end product. However, the fate of cytosolic MACC is less sure, and perhaps MACC can migrate to other plant parts (Bouzayen *et al*., 1988). There is some evidence that MACC is transported over longer distances, as basipetal transport of MACC was suggested in wounded pea (*Pisum sativum*) plants (Fuhrer & Fuhrer‐Fries, 1985) and MACC has been retrieved in the phloem of pumpkin (*Cucurbita pepo*) after an application with the synthetic auxin 1‐naphthylacetic acid (Amrhein *et al*., 1982). However, another study in cotton (*Gossypium hirsutum*) plants only observed phloem‐mediated ACC, and not MACC transport (Morris & Larcombe, 1995). Finlayson *et al*. (1991) retrieved MACC in the xylem of sunflower (*Helianthus annuus*) seedlings that were treated with exogenous ACC. Altogether, more research is needed to create clarity about long‐distance MACC transport and the existence of dedicated transporters.

### GACC: apoplastic conjugation of ACC

A second derivative, GACC, was found to be present in much lower amounts compared with MACC levels in mature green tomato fruit (Martin *et al*., 1995). GACC is made from glutathione (γ‐Glu‐Cys‐Gly) and ACC by γ‐glutamyl transpeptidase (GGT) (Martin *et al*., 1995; Martin & Slovin, 2000). GGT has been purified from different species such as tomato, onion, radish and Arabidopsis (Martin & Slovin, 2000; Shaw *et al*., 2005; Nakano *et al*., 2006). In Arabidopsis, four *GGT* genes (*GGT1‐4*) have been identified, of which only GGT1 and GGT2 appear to be catalytically active (Martin *et al*., 2007). GGT1 accounts for roughly all the activity to form GACC, while GGT2 activity is mainly restricted to seeds (Martin *et al*., 2007). GACC formation also influences ethylene‐mediated responses, as *ggt1* knockout mutants showed premature leaf senescence (Martin *et al*., 2007). A possible explanation for this premature senescence could be that the pool of ACC is replenished and more ACC is available to form ethylene. Suprisingly, both GGT1 and GGT2 seem to be localised extracellularly, suggesting that GACC is formed in the apoplast (Martin *et al*., 2007). This observation made us wonder about the exact apoplastic role of ACC conjugation into GACC. Perhaps extracellular GACC production serves to prevent apoplastic ACC to enter the cell, or to sequester ACC out of the cell, in order to regulate ethylene biosynthesis. Alternatively, GACC itself could be involved in cell wall signalling or sensing early stress responses, or it has a predominant role in the glutathione metabolism of plants.

### JA‐ACC: on the crossroads between ethylene and jasmonic acid signalling

In 2004, the newest derivative of ACC was identified as JA‐ACC in Arabidopsis, which is made by JAR1 from jasmonic acid and ACC (Staswick & Tiryaki, 2004). JAR1 plays an important role in activating JA to JA‐isoleucine and other JA‐amido conjugates including the nonproteinogenic amino acid ACC (Staswick & Tiryaki, 2004). Suprisingly, JA‐ACC levels were twice as high in *jar1* mutants compared with wild‐type plants, suggesting that JAR1 negatively regulates JA‐ACC levels or other JA‐ACC conjugating enzymes exist (Staswick & Tiryaki, 2004). The real function of JA‐ACC remains elusive, but it has been shown to inhibit root growth in Arabidopsis (Staswick & Tiryaki, 2004). Using JA and ethylene insensitive mutants, *coi1‐35* and *etr1‐1* respectively, it was shown that the inhibitory effect of JA‐ACC on root growth was accounted for by ethylene and not JA (Staswick & Tiryaki, 2004). Perhaps JA‐ACC is involved in hormonal crosstalk during biotic stress, as both ethylene and JA play an important role in plant defence to necrotrophic pathogens (Li *et al*., 2019).

## The regulation of ACC homeostasis via ACC transport

In addition to conjugation, transport of ACC plays an important role in controlling the spatial distribution of ethylene biosynthesis, as described in recent reviews (Vanderstraeten & Van Der Straeten, 2017; Polko & Kieber, 2019). Because ethylene rapidly diffuses as a gas, dedicated ACC transport enables remote ethylene signalling within plants. Both short‐ and long‐distance transport of ACC has been known for decades. Bradford & Yang (1980) observed xylem‐mediated root‐to‐shoot transport of ACC during root hypoxia of waterlogged tomato plants. While the major transport route of ACC is likely to be mediated by the xylem, ACC transport via the phloem has also been observed (Amrhein *et al*., 1982; Morris & Larcombe, 1995). In addition to long‐distance transport via vascular tissues, short distance transport of ACC was demonstrated in soybean (*Glycine max*) which showed a reduced ACC uptake when fed neutral amino acids (Lurssen, 1981). These early observations suggested the existence of a dedicated amino acid transporter that can also mobilise ACC. Additionally, intracellular transport of ACC across the tonoplast into the vacuole has also been demonstrated (Tophof *et al*., 1989; Saftner & Martin, 1993). It was decades later that the first ACC transporter was identified by Shin *et al*. (2015), who discovered an Arabidopsis mutant that was insensitive to ACC, but showed a normal response to gaseous ethylene. This mutant, designated *ACC‐resistant2* (*are2*), harbours a functional disruption of the amino acid transporter LYSINE HISTIDINE TRANSPORTER1 (LHT1) (Shin *et al*., 2015). LHT1 was previously identified as a transporter for positively charged amino acids (histidine, lysine and arginine) in plant roots (Chen & Bush, 1997; Hirner *et al*., 2006), confirming older speculations that ACC and other amino acids would share a transporter (Lurssen, 1981). Recently, a second ACC transporter (LHT2) was identified by complementation of the Arabidopsis *lht1* ACC insensitive line (Choi *et al*., 2019). ACC transport activity of both LHT1 and LHT2 was confirmed by electrophysiological analysis of *Xenopus* oocytes expressing *LHT1* and *LHT2*. As LHT2 is mainly expressed in floral organs, and higher order mutants of ACS are known to show floral organ defects unrelated to ethylene signalling (Tsuchisaka *et al*., 2009), the lack of these defects seen in the *lht2* mutant led the authors to speculate that there may be other ACC transporters (Choi *et al*., 2019). The identification of new ACC transporters awaits new discoveries in ACC mobility and homeostasis.

## Regulation of ethylene biosynthesis by d‐amino acids

Early on, it was discovered that methionine is the general precursor of ethylene biosynthesis and that besides l‐methionine, d‐methionine can also stimulate ethylene production in apple discs (Lieberman *et al*., 1966). d‐Amino acids are stereoisomers and often enantiomers of l‐amino acids, which means they have very similar physical properties, however they can have different biological functions. d‐Amino acids are frequently encountered in high concentrations in the rhizosphere due to microbiological decay, and are readily taken up and metabolised by plants (Vranova *et al*., 2012).

In general, l‐amino acids inhibit the production of ethylene when exogenous ACC is fed (Lurssen, 1981; Liu *et al*., 1983). This observation hinted at the competitive inhibition in uptake between ACC and other l‐amino acids, which was confirmed, as LTH1 and LTH2 can both transport ACC and other l‐amino acids such as l‐proline, l‐lysine, l‐histidine and l‐arginine (Hirner *et al*., 2006; Shin *et al*., 2015; Choi *et al*., 2019). By contrast, d‐amino acids were shown to stimulate ethylene production in several plant species (Liu *et al*., 1983; Kionka & Amrhein, 1984). An older hypothesis suggested that d‐amino acids compete with ACC for N‐malonylation by AMT (Ogawa *et al*., 1973; Kawasaki *et al*., 1982). Several laboratories found that d‐amino acids inhibit the formation of MACC when feeding exogenous ACC (Liu *et al*., 1983, 1984; Kionka & Amrhein, 1984; Su *et al*., 1985; Benichou *et al*., 1995; Chick & Leung, 1997). As such, the presence of d‐amino acids results in less MACC formation, consequently leading to higher ACC and ethylene levels. This concept is further corroborated by the observation that *in vivo* malonylation of d‐amino acids was stimulated by ethylene treatment (Liu *et al*., 1985b), similar to how ethylene can stimulate MACC formation by AMT (Martin & Saftner, 1995). This might suggest that AMT has a prime role in d‐amino acid metabolism and not in ACC sequestering. However, it is also possible that there are multiple specialised malonyltransferases, which have selected specificities towards d‐amino acids and ACC. This hypothesis is supported by the observation that 18 different d‐amino acids did not inhibit the activity of a partially purified AMT (Martin & Saftner, 1995). Furthermore, it was shown that ACC is not a substrate for other amino acid malonyltransferases (Wu *et al*., 1995). We believe that multiple specialised enzymes exist to process d‐amino acids and ACC. This theory is supported by a recent observation in Arabidopsis, in which d‐amino acid transaminase 1 (DAT1) was shown to have a specialised role in d‐amino acid metabolism (Suarez *et al*., 2019). DAT1 is able to transaminate d‐methionine into d‐alanine, d‐glutamic acid and l‐methionine. The *dat1* loss of function mutant showed an increased d‐amino‐acid‐stimulated ethylene production compared with wild‐type plants when fed d‐methionine (Suarez *et al*., 2019). The authors also showed that this increase in ethylene production is not caused by a reduced malonylation of ACC, as both malonylated d‐methionine (malonyl‐d‐methionine) and ACC (MACC) levels were higher in *dat1* mutants (Suarez *et al*., 2019). In conclusion, it appears that there are specialised enzymes that metabolise d‐amino acids and conjugate ACC, but also that there are still unknown biochemical links that could explain the d‐amino acid stimulated ethylene production in plants.

## Conclusions and outstanding questions

Despite the fact that the ethylene biosynthesis pathway is a simple two‐step pathway and was discovered 4 decades ago, its regulation is less well understood. Recently the identity of several transcription factors that control *ACS* and *ACO* gene expression have been unravelled, with many more likely to be uncovered. Both enzymes also show a complex posttranslational regulation, of which the stability and dimerisation of ACS has been thoroughly studied. However, the role and biological significance of the redox‐mediated posttranslational regulation of ACO remains elusive. At the metabolic level, it is clear that SAM levels and the activity of the Yang cycle play an important role in sustaining ethylene production. The pleiotropy of other SAM‐demanding metabolic processes and the multistep recycling pathway of MTA, create many additional layers of complexity that influence the regulation of SAM homeostasis. On top of that, our knowledge of the regulation of the ACC pool is far from clear. Despite the identification of three ACC conjugates and two ACC transporters, the molecular regulation of these ACC‐dependent steps are unknown and could advance the field. The emerging role of ACC as an ethylene‐independent growth regulator only strengthens the belief that ACC levels are tightly controlled. The dual function of ACC in signalling and ethylene biosynthesis will certainly boost novel discoveries that will shed more light on ACC formation, conjugation, degradation and localisation.

## Author contributions

JP, JV‐H and BVDP performed the literature research and wrote the paper. JP and JV‐H contributed equally to this work.
